# Formononetin triggers ferroptosis in triple-negative breast cancer cells by regulating the mTORC1/SREBP1/SCD1 pathway

**DOI:** 10.3389/fphar.2024.1441105

**Published:** 2024-09-27

**Authors:** Dong Xie, Yulang Jiang, Huan Wang, Lingyi Zhu, Shuangqin Huang, Sheng Liu, Weihong Zhang, Tian Li

**Affiliations:** ^1^ Shanghai Baoshan Hospital of Integrated Traditional Chinese and Western Medicine, Shanghai, China; ^2^ Shanghai University of Traditional Chinese Medicine, Shanghai, China; ^3^ Shuguang Hospital, Shanghai University of Traditional Chinese Medicine, Shanghai, China; ^4^ Yueyang Hospital of Integrated Traditional Chinese and Western Medicine, Shanghai University of Traditional Chinese Medicine, Shanghai, China; ^5^ General department, Songnan Town Community Health Service Center, Shanghai, China

**Keywords:** triple-negative breast cancer, ferroptosis, Formononetin, mammalian target of rapamycin complex1, sterol regulatory element-binding protein 1

## Abstract

**Introduction:**

Triple-negative breast cancer (TNBC) is the most malignant type of breast cancer, and its prognosis is still the worst. It is necessary to constantly explore the pathogenesis and effective therapeutic targets of TNBC. Formononetin is an active ingredient with anti-tumor effects that we screened earlier. The main purpose of this study is to elucidate mechanism of the inhibitory effect of Formononetin on TNBC.

**Methods:**

We conducted experiments through both *in vivo* and *in vitro* methodologies. The *in vivo* experiments utilized a nude mice xenotransplantation model, while the *in vitro* investigations employed two breast cancer cell lines, MDA-MB-231 and MDA-MB-468. Concurrently, ferroptosis associated proteins, lipid peroxide levels, and proteins related to the rapamycin complex 1 were analyzed in both experimental settings.

**Results:**

In our study, Formononetin exhibits significant inhibitory effects on the proliferation of triple TNBC, both *in vivo* and *in vitro*. Moreover, it elicits an increase in lipid peroxide levels, downregulates the expression of ferroptosis-associated proteins GPX4 and xCT, and induces ferroptosis in breast cancer cells. Concurrently, Formononetin impedes the formation of the mammalian target of rapamycin complex 1 (mTORC1) and suppresses the expression of downstream Sterol regulatory element-binding protein 1(SREBP1). The utilization of breast cancer cells with SREBP1 overexpression or knockout demonstrates that Formononetin induces ferroptosis by modulating the mTORC1-SREBP1 signaling axis.

**Discussion:**

In conclusion, this study provides evidence that Formononetin exerts an anti-proliferative effect on triple-negative breast cancer by inducing ferroptosis. Moreover, the mTORC1-SREBP1 signal axis is identified as the primary mechanism through which formononetin exerts its therapeutic effects. These findings suggest that formononetin holds promise as a potential targeted drug for clinical treatment of TNBC.

## 1 Introduction

According to data analysis from the International Agency for Research on Cancer (IARC) and the National Cancer Registration Center of our country, the incidence of breast cancer has been increasing steadily over the past 40 years, and it has consistently ranked first in the incidence of all cancers (Giaquinto et al., 2022). Triple-negative breast cancer (TNBC) is the most malignant type of breast cancer, accounting for approximately 10%–15% of all breast cancers. Currently, the main treatment for TNBC is chemotherapy, and in recent years, targeted therapy for different subtypes of TNBC has been added in the context of precision medicine, with an objective response rate of only 29% (Jiang Y. Z. et al., 2021). TNBC still has the worst prognosis among subtypes (Tsang and Tse, 2023). In the era of precision medicine, research and development of new drugs targeted at different treatment targets is ongoing, but currently, various targeted drugs for treating TNBC have not been approved by the FDA (Garrido-Castro et al., 2019; Hwang et al., 2019). Therefore, it is still necessary to continue exploring the pathogenesis of TNBC and effective treatment targets. Recent studies have found that ferroptosis, as a new type of cell death process, can enhance the treatment effect of breast cancer, especially in TNBC (Chen et al., 2021; Verma et al., 2020).

Ferroptosis, as a new type of cell death process, is different from traditional cell death pathways (such as apoptosis, necroptosis, necrosis, autophagy). Its characteristic is the accumulation of reactive oxygen species-induced lipid peroxidation. Multiple intracellular molecules regulate the onset of ferroptosis by influencing intracellular iron levels and lipid peroxidation status (Li et al., 2020; Xie et al., 2016; Zhou et al., 2024). Cells undergoing ferroptosis exhibit morphological features that are distinct from other programmed cell death modalities, such as apoptosis, autophagy, necroptosis, and pyroptosis. The most prominent morphological changes in ferroptosis include mitochondrial atrophy and reduction of cristae, while the nucleus remains intact. Ferroptosis can be induced by structurally distinct small molecules, including erastin, sulfasalazine, and RSL3, or inhibited by lipophilic antioxidants such as CoQ10, vitamin E, ferrostatins, and liproxstatins. Elevated intracellular iron ion concentrations and deficiency of the antioxidant GSH lead to cellular lipid peroxidation, ultimately resulting in ferroptosis.

The principal regulatory mechanisms of ferroptosis involve lipid peroxidation, amino acid metabolism, oxidative stress, iron ion homeostasis, and various organelles, all of which are prerequisites for the occurrence of ferroptosis. The body naturally possesses a ferroptosis defense system to protect cells, which primarily includes 1) Cystine/System Xc−/GSH/GPX4, 2) FSP1-CoQ10-NAD(P)H, 3) DHODH-CoQ10-CoQH2, 4) GCH1/BH4/DHFR, and 5) FSP1/ESCRT-III (Jiang and Sun, 2024). The body maintains a dynamic equilibrium of cellular state by modulating both the promotive and resistive factors of ferroptosis (Wang et al., 2023). These essential prerequisites and defense mechanisms inherently suggest that ferroptosis is a metabolic form of cell death (Jiang et al., 2024a). While the physiological relevance of ferroptosis remains a subject of debate, its undeniable connection to TNBC is well-established (Jiang et al., 2024b).

There is hope that increasing ferroptosis in TNBC could improve the effective treatment rate. However, most ferroptosis-related drugs for breast cancer are still in the preclinical stage (Lin et al., 2021). Although some marketed drugs have been proven to be related to ferroptosis, the relevant trial results have not been published. However, these trials include patients with HER-2 positive breast cancer and bone metastasis, and there are few clinical trials specifically targeting TNBC. Additionally, these drugs are usually used in combination with chemotherapy, which may increase the burden on liver and kidney function. Therefore, further screening of compounds that can promote ferroptosis from natural products to enhance the efficacy of TNBC could be considered.

Formononetin, an isoflavone compound derived from a variety of medicinal plants and herbs such as including *Trifolium pratense* and *Astragalus membranaceus*, has been identified as exhibiting anticancer potential against various cancer types, such as cervical, head and neck, colon, breast and ovarian cancers (Almatroodi et al., 2023; Almatroodi et al., 2023). In our previous research, formononetin was found to exhibit anti-triple-negative breast cancer properties through the enhancement of the tumoricidal effect of everolimus *via* the inhibition of Akt activity (Li et al., 2021; Zhou et al., 2019), indicating its potential as a therapeutic agent for breast cancer inhibition.

Given the significant role of ferroptosis in tumor-related mortality, our study investigates the novel approach of formononetin in inducing cell death in triple-negative breast cancer. Our aim is to ascertain if formononetin can induce ferroptosis and elucidate the potential anti-tumor mechanism.

## 2 Experimental procedures

### 2.1 Materials and reagents

Experimental materials and reagents are displayed in Supplementary Table S1.

### 2.2 Animal handling

SPF grade female BALB/c nude mice (weighing 17 ± 3 g) were purchased from Slaccas animal Co. Ltd. (Shanghai, China), raised in the Experimental Animal Center of Shanghai University of Traditional Chinese Medicine, and maintained under a 12-h- light/dark cycle with free access to food and water.

After adaptive feeding for 3 days, the model was constructed. MDA-MB-231 cells in logarithmic growth phase were subcutaneously inoculated on the dorsal region of nude mice. A suspension of 1 × 10^6^ cells in a 1:1 mixture of PBS and Matrixgel was utilized to establish tumor implantation models in each nude mouse. Upon reaching a volume of 100 mm^3, the mice with xenograft were randomly allocated into different groups for medication intervention.

The mice were randomly divided into four groups (n = 6/each group): Control group, Formononetin group (50 mg/kg), RSL3 group (50 mg/kg), Paclitaxel group (50 mg/kg). All pharmacological agents were formulated as suspensions for *in vivo* administration to animals, utilizing a vehicle system composed of 10% dimethyl sulfoxide, 40% polyethylene glycol 300 (PEG300), 5% Tween-80, and 45% saline. The administration of all drugs was performed *via* gavage, whereas the control group received an equivalent volume of physiological saline by the same method. Follow up testing was conducted after a total of 28 days of administration.

### 2.3 Cell culture

MDA-MB-231 and MDA-MB-468 human breast cancer cell lines were purchased from the Cell Bank of the Chinese Academy of Sciences. Cells were subcultured in DMEM supplemented with 10% FBS and 1% Penicillin-Streptomycin solution to facilitate subsequent experimental procedures.

### 2.4 Cell proliferation assay

The effect of formononetin on cell proliferation ability was determined by CCK8 assay and plate clone formation assay. Briefly, A cell suspension with a cell density of 5,000 cells *per* well was prepared and incubated in a 96 well plate for a specific time period. Add 10 μL of CCK8 solution to each well and allow the cck8 reagent to react with viable cells for 4 h. Measure the absorbance of the pores using a microplate reader at a wavelength of 450 nm. The cell viability was calculated by comparing the absorbance values of the treated cells with those of the control cells. The plate clone formation experiment was carried out according to the following steps. A cell suspension with a cell density of 1,000 cells *per* well was cultured in a 6-well plate for 48 h, and corresponding drugs were added for intervention to form colonies. After this, fix and use crystal violet staining solution for cell colony staining. A microscope was used for counting and analyze the form colonies.

### 2.5 Lipid peroxide detection

The effect of Formononetin on cell proliferation ability was determined by liperfluo, MDA and GSH assay kits. Cellular lipid peroxidation was assessed using Liperfluo (One day before treatment, 4 × 10^5^ cells/well were seeded in 6-well plates. Cells were harvested 24 h after different treatments. After incubation with 1 μM Liperfluo for 30 min, the cells were measured by flow cytometry. The percentage of the number of FITC channel-positive cells in each group of cells was analyzed using flowjo software. The percentage of positive cells in each group was statistically analyzed as the percentage of the total number of cells above a specific threshold fluorescence intensity., and MDA and GSH were detected according to the reagent instructions.

### 2.6 Reactive oxygen species assay

A cell suspension with a cell density of 2*10^6 was cultured in a 12 well plate, and the corresponding drug intervention was scheduled the next day. After incubation with reactive oxygen species fluorescent probes for 1 h, the cells were washed with PBS to remove excess probes. Fluorescence microscopy was used to analyze fluorescence signals to evaluate the levels of reactive oxygen species (ROS) in cells.

### 2.7 Iron assay

A total of 100 mg of tissue was subjected to sonication and subsequent centrifugation to obtain the supernatant, which was then utilized for the determination of Fe^2+^ concentration. Following the guidelines provided by the reagent kit, the corresponding Reduce solution and Assay Buffer were added to the supernatant and incubated at a temperature of 37°C for a duration of 15 min. The resulting mixture was transferred to a 96 well plate and incubated for 60 min. Subsequently, the absorbance at a wavelength of 593 nm was measured, and the corresponding Fe^2+^ concentration was calculated based on the established standard curve.

### 2.8 Immunofluorescent staining

Xenograft tissues were fixed with 4% paraformaldehyde and embedded in paraffin. Then they were cut into 4-μm thick coronal brain sections and mounted on slides. Antigen repair was performed in sodium citrate buffer at 95°C to 100 °C for 40 min. Correspondingly, in term of cell samples, they were fixed with 4% paraformaldehyde for 40 min before proceeding to the next step of the experiment. After blocking with 10% goat serum at room temperature, sections were incubated with primary antibody at 4°C overnight. Sections were washed with Tris-buffered saline (TBS) and incubated with secondary antibody anti-mouse-IgG antibody conjugated with Alexa Fluor488, anti-rabbit-IgG antibody conjugated with Alexa Fluor 594 for 2 h at room temperature in the dark. Cell nucleus was stained with DAPI for 15 min at room temperature away from light. Images were acquired with confocal microscope. The results from the three sections were averaged to reflect the level of protein expression.

### 2.9 Western blotting

RIPA lysate buffer supplemented with proteinase and phosphatase inhibitors was employed to disrupt cells and tissues. The protein concentration of the resulting lysates was determined using the BCA method. Subsequently, the protein samples were mixed with SDS-PAGE loading buffer and subjected to boiling at 100°C for a duration of 10 min. Western blotting experiments were subsequently conducted. Briefly, after sampling 20 μg of protein, electrophoresis was performed to separate the proteins, which were then transferred onto a PVDF membrane. The membrane was subsequently blocked with 5% BSA for a period of 1 h. Subsequently, the protein membranes were subjected to an overnight incubation at 4°C with the primary antibody. Following membrane washing, the corresponding secondary antibodies were incubated for 2 h at room temperature. The protein bands were acquired through ECL chemiluminescence and quantified and standardized using ImageJ software v1.8.0.

### 2.10 Immunohistochemistry

The immunohistochemistry (IHC) procedure involves several steps. First, the tumor tissues were fixed and embedded. Then, it is cut into thin sections measuring 4 μm. Antigen repair was performed in sodium citrate buffer at 95°C to 100°C for 40 min. To minimize non-specific binding, 5% BSA was used for blocking. The primary antibody was incubated at 4°C overnight to facilitate specific binding of the target antigen. Subsequently, the secondary antibody, conjugated with a detection molecule, was incubated to bind to the primary antibody. The visualization of target proteins was achieved through the utilization of DAB colorimetric solution. Subsequently, a quantitative analysis of each sample was conducted from three distinct perspectives.

### 2.11 Cell plasmid transfection

A cell suspension was prepared with varying cell densities in different well plates: 8,000 cells *per* well in a 96-well plate, 8*10^5 cells *per* well in a 12-well plate, and 2*10^6 cells *per* well in a 6-well plate. The cells were incubated for 24 h. For transfection, 2.5 μg DNA *pe*r well in a 6-well plate, 1 μg DNA *per* well in a 12-well plate, 100 ng DNA *per* well in a 96-well plate were utilized. The plasmid mixture was prepared by mixing the plasmid DNA with a Lipo8000™ transfection reagent in a 1:1.6 ratio, was then added into the cell plates and incubated for a duration of 24 hto allow the internalization of the plasmid DNA. Following transfection, subsequent drug intervention was conducted.

### 2.12 Observation of mitochondrial ultrastructure by transmission electron microscopy

The procedure for observing cellular samples using TEM involves fixing, dehydrating, embedding, sectioning, mounting, staining, and imaging. The cell structure was initially stabilized using a room temperature glutaraldehyde fixative, followed by dehydration using an alcohol solution with increasing gradient concentration. Subsequently, the cells were embedded in resin to provide structural support and maintain their ultrastructure. The embedded cells were then thinly sliced, approximately 50–100 nm thick, using an ultra-fine slicer and mounted on a grid typically composed of copper or nickel. To enhance contrast and visualize structural details, the mounting grid was stained with heavy metals such as uranyl acetate.

### 2.13 Statistical analysis

After the experiment data using GraphPad Prism 8.0 software for statistical analysis, all data were expressed as mean (Mean) ± standard deviation (SD) in statistics. Statistical differences in the data were analyzed by One-way or Two-way analysis of variance (ANOVA).

### 2.14 Statement of ethics

The procedures for care and use of animals were approved by the Ethics Committee of Shanghai University of Traditional Chinese Medicine, and all applicable institutional and governmental regulations concerning the ethical use of animals were followed.

## 3 Results

### 3.1 Formononetin suppress breast cancer cell proliferation through ferroptosis

**FIGURE 1:**
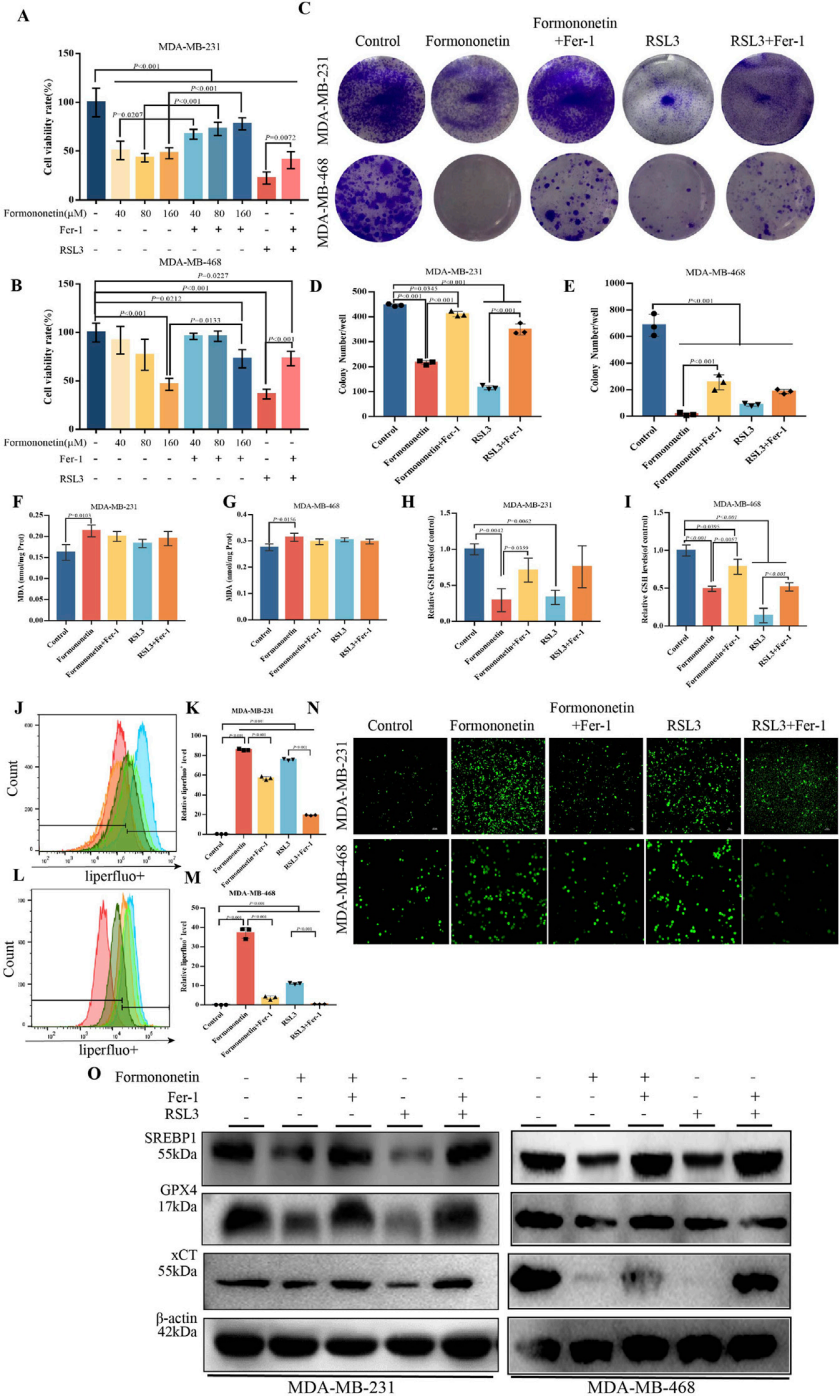
Formononetin can inhibit the proliferation of triple-negative breast cancer cells and induce ferroptosis. **(A)**. The cell viability of MDA-MB-231 treated with Formononetin after 48 h s of intervention at 40 μM, 80 μM, 160 μM, 1 μM RSL3, and 10 μM Fer-1 for 24 h was measured by using cck8 method. **(B)**. The cell viability of MDA-MB-468 treated with Formononetin after 48 h of intervention at 40 μM, 80 μM, 160 μM, 1 μM RSL3 and 10 μM Fer-1 for 24 h was measured using cck8 method. The drug intervention protocols employed on MDA-MB-231 and MDA-MB-468 cells for subsequent experiments are outlined as follows:160 μM Formononetin for 48 h, 10 μM Fer-1 for 24 h, 1 μM RSL3 for 24 h. **(C–E)**. Plate colony formation experiments and quantity analysis on MDA-MB-231 and MDA-MB-468 cells. **(F, G)** MDA content in cell lysates of MDA-MB-231 and MDA-MB-468 cells. **(H, I)**. The expression levels of GSH relative to the control in MDA-MB-231 and MDA-MB-468 cells. **(J–M)** Detection of liperfluo by flow cytometry and quantitative analysis of relative expression levels on MDA-MB-231 and MDA-MB-468 cells. **(N)** The level of ROS in MDA-MB-231and MDA-MB-468 cells. **(O)** The levels of expression of ferroptosis-related proteins. Data are reported as mean ± SD. *P* values are indicated in figure. The quantity analysis was performed on three independent samples. (MDA, malondialdehyde, GSH, glutathione, Fer-1, Ferrostatin-1, ROS, reactive oxygen species).

In order to ascertain the potential anticancer effects of Formononetin, breast cancer cell lines MDA-MB-231 and MDA-MB-468 were selected for CCK8 experiments to assess the impact of Formononetin treatment on cell proliferation. Upon intervention with Formononetin, the viability of both MDA-MB-231 and MDA-MB-468 cells exhibited a dose-dependent decrease (Figures 1A, B). Notably, Formononetin treatment significantly impeded the proliferation capacity of breast cancer cells, as evidenced by the inhibition of colony formation observed in clone formation assays. (Figures 1C–E). In order to ascertain the specific modes of cell death induced by Formononetin, Fer-1, a targeted ferroptosis inhibitor, was utilized to investigate the cell death response of MDA-MB-231 and MDA-MB-468 cells. Additionally, we employed RSL3, a well-established ferroptosis inducer, to assess the potential of Formononetin as a novel ferroptosis inducer. Our findings indicate that under 160 μM formononetin treatment, Fer-1 was able to reverse the cell death and clone formation observed in MDA-MB-231 and MDA-MB-468 cells. Furthermore, the positive control, RSL3, demonstrated a significant ferroptosis effect (Figures 1A–E). Based on the aforementioned information, we formulated the hypothesis that Formononetin may serve as a novel inducer of ferroptosis. Ferroptosis typically arises from the intracellular accumulation of lipid peroxides and a decline in antioxidant level (Dixon,et al., 2012). Consequently, we proceeded to investigate the alterations in MDA content and GSH content in MDA-MB-231 and MDA-MB-468 cells upon the administration of Formononetin. Additionally, we assessed the response to this intervention by evaluating the impact of Fer-1. The findings revealed that Formononetin exhibited the ability to enhance the MDA content in both MDA-MB-231 and MDA-MB-468 cells, with a more pronounced effect observed compared to RSL3 (as depicted in Figures1F, G). In our study, it was observed that Formononetin exhibited a significant reduction in intracellular GSH content, which was partially restored by the administration of Fer-1 (Figures 1H, I). Additionally, the content of specific lipid peroxides within MDA-MB-231 and MDA-MB-468 cells was assessed using a liperfluo probe. The flow peak plots demonstrated a significant increase in intracellular lipid peroxides induced by Formononetin, when compared to the control and RSL3 groups (Figures 1J–M). Moreover, the analysis of ROS fluorescence plots revealed that Formononetin resulted in a higher intensity of intracellular ROS levels compared to RSL3 fluorescence. Furthermore, it was observed that Fer-1 exhibited varying degrees of inhibition on the elevated levels of reactive oxygen species (ROS) (Figure 1N). This illustrates the ability of Formononetin to induce ferroptosis. Our findings further support the induction of ferroptosis through the detection of proteins xCT, GPX4, and SREBP1. Formononetin was found to downregulate the expression of these proteins within MDA-MB-231 and MDA-MB-468 cells, consistent with the effect of RSL3, which could be counteracted by Fer-1 (Figure 1O).

### 3.2 Formononetin inhibits breast cancer proliferation *in vivo*

**FIGURE 2:**
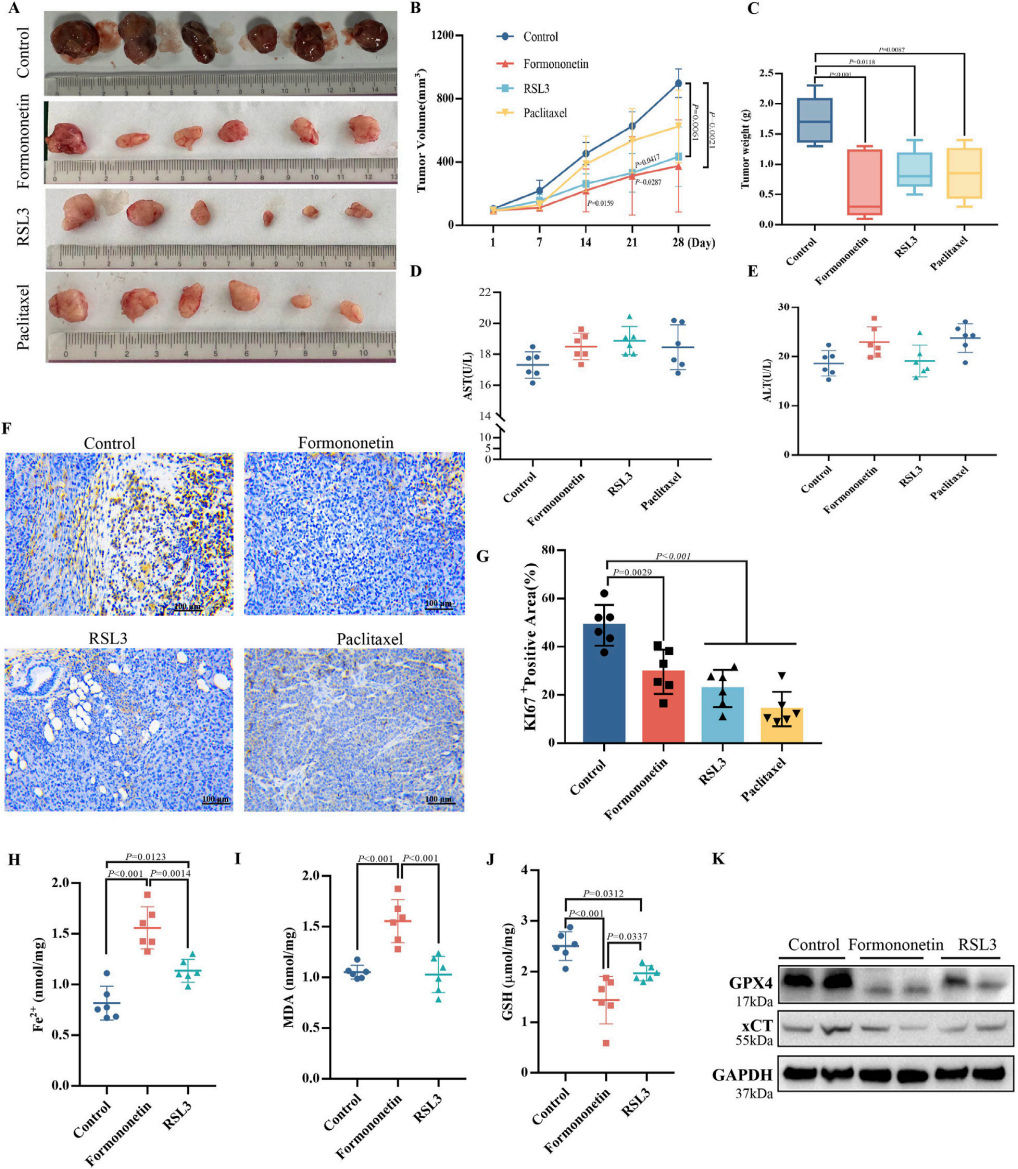
The inhibitory effect of Formononetin on the size and proliferation of xenografts in nude mice is associated with the induction of ferroptosis in breast cancer cells. **(A)** Visual image of xenograft of nude mice. **(B)** Tumor volume size of xenograft of nude mice. (Calculation formula = width^2^ x length/2) **(C)** Tumor weight xenograft of nude mice. **(D, E)** Expression levels of AST and ALT in the liver. **(F, G)** Immunohistochemical staining and quantitative analysis of ki67. **(H)** Fe^2+^ content in xenograft of nude mice. **(I)** GSH content in xenograft of nude mice. **(J)** MDA content in xenograft of nude mice. **(K)** The expression levels of ferroptosis-related proteins. Data are reported as mean ± SD. *P* values are indicated in figure. (MDA, malondialdehyde; GSH, glutathione; AST, Aspartate aminotransferase; ALT, Alanine aminotransferase).

In order to validate the *in vivo* anti-tumor efficacy of Formononetin, nude mice were subcutaneously injected with MDA-MB-231 cells to establish a mouse xenograft tumor model. Through the analysis of tumor images, as well as measurements of tumor size and time-volume, it was observed that mice treated with Formononetin exhibited significantly reduced subcutaneous tumor growth and lower final tumor weights compared to the control group. Furthermore, Formononetin demonstrated superior tumor inhibition when compared to RSL3, a ferroptosis inducer, and paclitaxel, a clinical chemotherapeutic drug (Figures 2A–C). The biomarker Ki67 was utilized to assess the proliferation of tumor cells (Cheang et al., 2009). Our findings indicate that the positive rate of Ki67 staining in paraffin sections was significantly lower in the Formononetin group compared to the control group (Figures 2F,G). Additionally, no abnormalities in diet and behavioral activities were observed in nude mice during the experiment, regardless of whether they were treated with or without Formononetin. We examined the serum levels of ALT and AST in mice and found that for had no impairment of liver function in mice (Figures 2D, E). This suggests that Formononetin exhibits good *in vivo* safety and does not pose significant toxicity to nude mice. Further investigation is warranted to confirm the potential of Formononetin in inducing ferroptosis *in vivo*. In this study, we conducted an examination of the levels of ferrous ions, GSH, and MDA content in tumors. We compared these levels with those of the control group and found that Formononetin exhibited a significant increase in Fe^2+^ and MDA content (Figures 2H, I), while also significantly decreasing GSH content (Figure 2J). Additionally, through further immunoblot analysis of tumor tissues, we observed that Formononetin treatment led to a reduction in the expression of intracellular ferroptosis-related proteins GPX4, and xCT (Figure 2K). Based on these findings, we can conclude that Formononetin has the potential to inhibit breast cancer proliferation both *in vitro* and *in vivo*, potentially through the pathway of ferroptosis.

### 3.3 Formononetin promotes ferroptosis through the mTORC1/SERBP1/SCD1 signaling pathway

**FIGURE 3:**
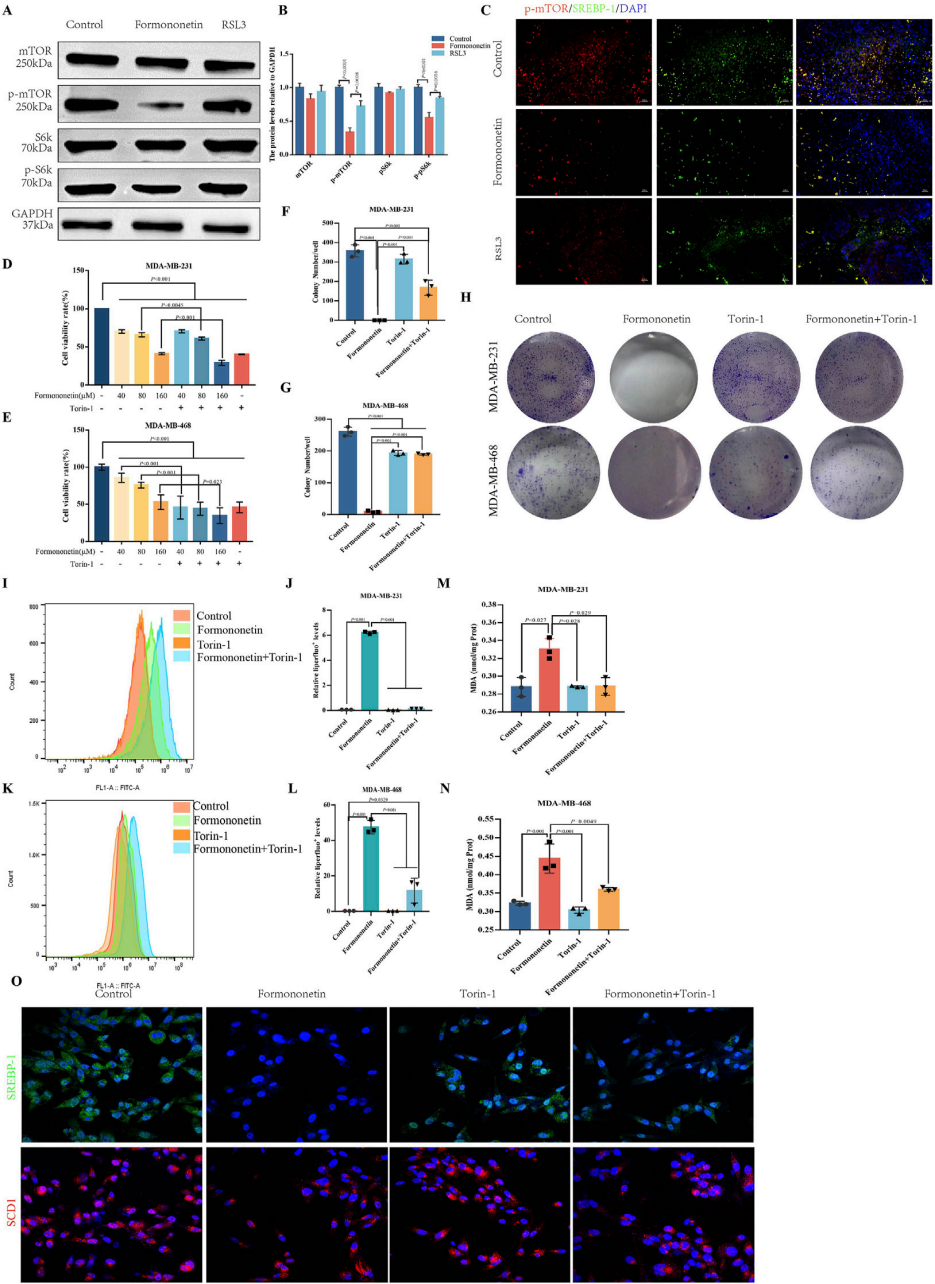
The induction of ferroptosis in breast cancer by Formononetin is inherently linked to the inhibition of mTORC1. **(A, B)** The protein expressions of mTOR, S6k and their phosphorylated proteins in xenograft tissues. Quantitative analysis uses three independent samples for grayscale value analysis. **(C)** Immunofluorescence staining of p-mTOR and SREBP-1 in xenograft tissues (red, p-mTOR,green,SREBP-1, blue, DAPI). **(D, E)** The cell viability of MDA-MB-231and MDA-MB-468 cells treated with Formononetin and mTORC1 inhibitor Torin-1 by cck8 method (drug concentration: 40 μM, 80 μM, 160 μM Formononetin for 48 h, 10 μM Torin-1 for 48 h). **(F–H)** Plate colony formation experiments on MDA-MB-231 and MDA-MB-468 cells (drug concentration: 160 μM Formononetin for 48 h, 10 μM Torin-1 for 48 h) were conducted, and colony formation quantity analysis was performed on three independent samples. **(I–L)** Detection of liperfluo by flow cytometry on MDA-MB-231 and MDA-MB-468 cells, quantitative analysis of relative expression levels using three independent samples. (drug concentration: 160 μM Formononetin for 48 h, 10 μM Torin-1 for 48 h s). **(M, N)** MDA content in cell lysates of MDA-MB-231 and MDA-MB-468 cells. (drug concentration: 160 μM Formononetin for 48 h, 10 μM Torin-1 for 48 h). **(O)** Immunofluorescence staining of SREBP1 and SCD1(drug concentration: 160 μM Formononetin for 48 h, 10 μM Torin-1 for 48 h). Data are reported as mean ± SD. *P* values are indicated in figure.

The mTOR signaling pathway plays a crucial role in cell growth, metabolism, and disease (Hua et al., 2019). It functions as a downstream signaling factor of PI3K/AKT and consists of two protein complexes, mTOR1 and mTOR2 (Porta et al., 2014; Zou et al., 2020). Through phosphorylation of the downstream SREBP1-SCD1, mTOR1 facilitates the synthesis of GPX4 and GSH, thereby enhancing the resistance of tumor cells to ferroptosis (Yi et al., 2020). Western blot analysis demonstrated that Formononetin significantly decreased the level of p-mTOR, p-pS6k, indicating that exposure to Formononetin could potentially impact the activity of the mTORC1 complex (Figures 3A, B). Immunofluorescence of the cells showed co-localization between SREBP1-chemical mTOR and that formononetin significantly inhibited the activity and binding of both. (Figure 3C). In order to provide additional evidence of the inhibitory effects of formononetin on breast cancer proliferation through its impact on mTORC1 activity, we conducted a co-administration study using Formononetin and the mTOR inhibitor Torin-1 on MDA-MB-231 and MDA-MB-468 cells. The results revealed that the combination of Formononetin and Torin-1, in comparison to Formononetin alone, effectively reversed the decrease in cell viability and clone formation (Figures 3D–H). Given the positive correlation observed between mTOR activity and downstream GSH content and lipid peroxide accumulation (Sekhar et al., 2022), we conducted a comparative analysis to examine the impact of Formononetin on downstream lipid peroxide, MDA, and GSH content in the presence or absence of Torin-1. The findings revealed that Torin-1 partially mitigated the elevation in intracellular lipid peroxide and MDA content, as well as the reduction in GSH content induced by formononetin (Figures 3I–N). Additionally, cellular immunofluorescence analysis demonstrated that formononetin decreased the expression of SCD1 and SREBP1, while intervention with Torin-1 resulted in increased expression levels of SCD1 and SREBP1 (Figure 3O).

### 3.4 Overexpression of SREBP1 attenuates formononetin-mediated ferroptosis sensitivity

**FIGURE 4:**
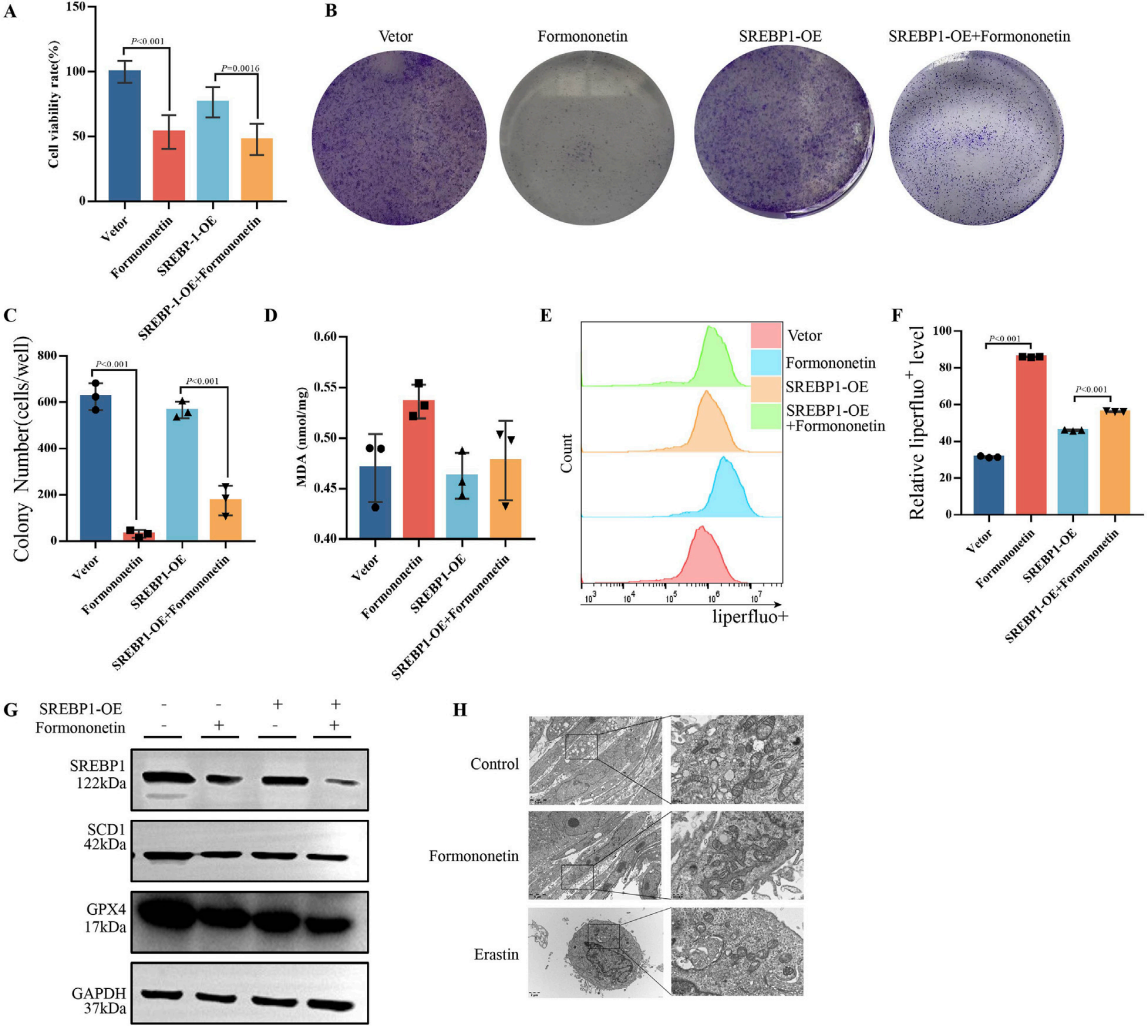
Formononetin induces ferroptosis in MDA-MB-231 cells by inhibiting SREBP1. MDA-MB-231 cells with or without SREBP1-overexpression plasmid transfection treated with 80 μM Formononetin for 48 hs. **(A)** The cell viability of MDA-MB-231 cells was measured by using cck8 method. **(B, C)** Plate colony formation experiments on MDA-MB-231 cells. **(D)**. MDA content in cell lysates of MDA-MB-231 cells. **(E, F)** Detection of liperfluo by flow cytometry on MDA-MB-231 cells. **(G)** Western blotting of the protein expressions of GPX4, xCT, SREBP1 in MDA-MB-231 cells. **(H)** Electron microscopy of cellular mitochondrial structure under Formononetin and Erastin intervention. Data are reported as mean ± SD. *P* values are indicated in figure.

To comprehend the fundamental objective of Formononetin intervention in mTORC1, we generated an overexpression plasmid and an empty plasmid of SREBP1, which were subsequently transfected into MDA-MB-231 cells. Following this, we observed the intracellular ferroptosis related biochemical indicators and proliferation in the presence or absence of Formononetin intervention. Our findings revealed that SREBP1 overexpression significantly attenuated the formononetin-mediated inhibition of breast cancer cell viability, and there was a reversion of cell viability (Figure 4A). The Clone Formation assay also indicated that overexpression of SREBP1 attenuated the inhibition of breast cancer cell clone formation caused by formononetin (Figures 4B, C). Our findings revealed that the overexpression of SREBP1 resulted in a significant reduction in intracellular lipid peroxides and MDA levels, while concurrently increasing the intracellular GSH content, when compared to the group treated with formononetin alone (Figures 4D–F). Additionally, Western blot analysis demonstrated a significant restoration of ferroptosis-related proteins GPX4 and xCT following SREBP1 overexpression (Figure 4G). These experimental results provide evidence supporting our hypothesis that SREBP1 serves as a central target in formononetin-induced ferroptosis. Ferroptosis, characterized by distinct cellular morphological features, prompted the utilization of electron microscopy to examine the mitochondrial morphology across different treatment group. Notably, the control group exhibited normal mitochondrial morphology with intact membranes (Jiang X. et al., 2021). Conversely, the formononetin group displayed evident mitochondrial wrinkling, augmented mitochondrial membrane density, and reduced cristae, mirroring the manifestations observed in the erastin group (Figure 4H). In summary, our study provides preliminary evidence that the main downstream target of Formononetin affecting mTOR complex activity is SREBP1.

### 3.5 SREBP1-KO inhibit ferroptosis mediated by formononetin

**FIGURE 5:**
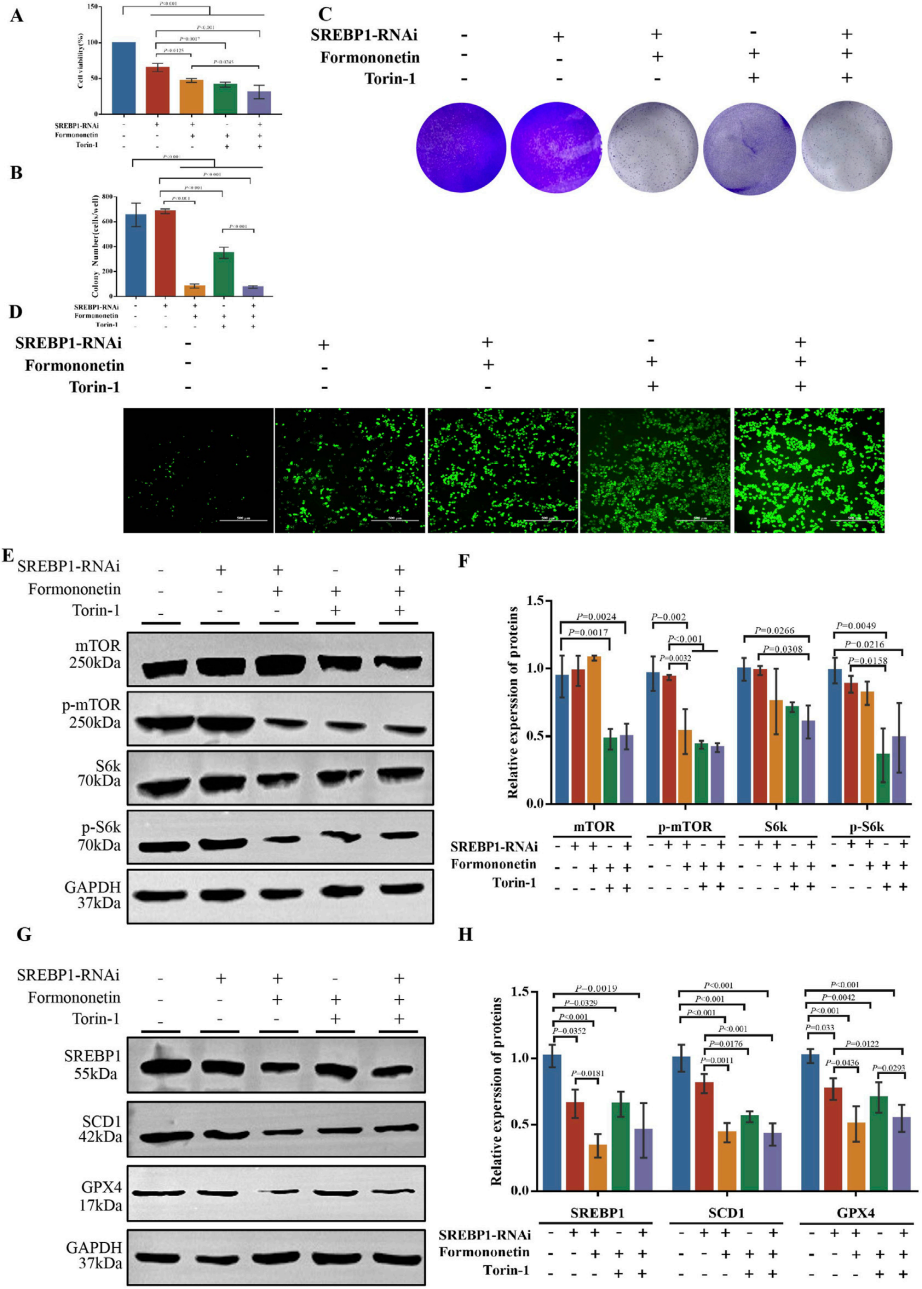
The inhibition of the proliferation of TNBC and the induction of ferroptosis by Formononetin are attributed to the negative regulation of the mTORC1-SREBP1 signal axis. MDA-MB-231 cells with or without SREBP1-RNAi plasmid transfection treated with 80 μM Formononetin for 48 h and/or 10 μM Torin-1 for 48 h. **(A)** The cell viability of MDA-MB-231 cells was measured by using cck8 method. **(B, C)** Plate colony formation experiments on MDA-MB-231 cells. **(D)**. The level of ROS in MDA-MB-231 cells. **(E, F)** Western blotting of the protein expressions of mTOR, p-mTOR, S6k, p-S6k in MDA-MB-231 cells. **(G, H)** Western blotting of the protein expressions of GPX4, SCD1, SREBP1 in MDA-MB-231 cells. Data are reported as mean ± SD. *P* values are indicated in figure.

To further substantiate the assertion that SREBP1 is a downstream target of Formononetin impeding mTOR activity, we established multiple experimental groups including SREBP1 RNA interference group, with or without Formononetin groups, and with or without Trion-1 groups. Initially, the CCK8 method was employed to assess the impact of formononetin and Torin-1 on the viability of the MDA-MB-231 cells in the presence of SREBP1 interference. Consistent with previous results, formononetin and Torin-1 led to a significant decrease in MDA-MB-231 cell viability. Additionally, SREBP1 interference also resulted in reduced cell viability, and the combined intervention of formononetin further enhanced this inhibitory effect. However, under conditions of SREBP1 silencing, the conditional intervention of Torin-1 did not reverse the decline in cell viability induced by formononetin (Figures 5A). The clonal formation assay demonstrated that formononetin, under conditions of SREBP1 silencing, attenuated the clonogenic potential of breast cancer cells, whereas Trion-1 did not rescue the clonogenic ability inhibited by formononetin (Figures 5B, C). Furthermore, measurement of intracellular ROS levels revealed that supplementation of formononetin led to elevated levels of ROS in SREBP1-RNAi MDA-MB-231 cells, whereas co-administration of Trion-1 did not mitigate ROS levels (Figure 5D). Western blot results showed that silencing SREBP1 did not rescue the inhibition of mTOR activity caused by Formononetin, as Trion-1 mediated downstream expression of ferroptosis inhibitors SCD1 and GPX4 (Figures 5E–H). This part of the experimental evidence confirms that SREBP1 is the target of Formononetin blocking mTORC1.

## 4 Discussion

Breast cancer has been identified as the most prevalent form of malignancy among females. TNBC is characterized by immunohistochemical findings indicating less than 1% presence of estrogen and progesterone receptors, as well as a negative HER-2 gene status. Despite the utilization of comprehensive treatment approaches for TNBC patients, the limited survival advantage can be attributed to its heightened invasiveness, significant heterogeneity, substantial tumor burden, and unfavorable prognosis (Siegel,et al., 2023). Consequently, there is an imperative need to explore novel targets and medications within the realm of TNBC treatment (Vagia,et al., 2020). Numerous studies have been conducted to investigate the therapeutic approaches for early-stage breast cancer, and inhibition of the PI3K/AKT/mTOR pathway has shown promising results (Cocco,et al., 2022; Zhu et al., 2023). Most importantly, the combination of mTORC1 inhibition and ferroptosis induction has shown therapeutic potential in preclinical models (Yi,et al., 2020).

The PI3K/AKT/mTOR signaling pathway is a prevalent mechanism of cancer activation, contributing to tumor cell proliferation and various malignant biological processes, as well as influencing the expression of genes related to cancer cell proliferation and apoptosis. Activation of the PI3K/AKT/mTOR pathway is commonly observed in TNBC, often due to gain-of-function mutations in PI3KCA and loss of function in PTEN (Cocco et al., 2020). Inhibition of the PI3K/AKT/mTOR signaling pathway has emerged as a significant approach to sensitizing cancer cells to Ferroptosis, a novel pathway of programmed cell death (Li et al., 2023). Ferroptosis, a form of programmed cell death (PCD), is characterized by dysregulation in ion metabolism, glutathione GSH metabolism, and lipid metabolism, leading to an imbalance in the body’s antioxidant system (Hirschhorn and Stockwell, 2019; Ong et al., 2019; Tian et al., 2022). Extensive research has established a strong association between ferroptosis and various challenging clinical conditions, including malignant tumors, neurodegenerative diseases, and acute kidney injury. Consequently, modulating the occurrence of ferroptosis holds promise for effective interventions in the diseases (Chen et al., 2021). The ongoing advancements in the investigation of the molecular mechanism. Concurrently, several investigations have demonstrated that various drug molecules can impede the proliferation of breast cancer cells by inducing ferroptosis, thereby suggesting that the induction of ferroptosis holds promise as a viable strategy for breast cancer treatment (Qiu et al., 2020; Su et al., 2022).

Formononetin, an isoflavone compound derived from Astragalus membranous, has been extensively studied for its diverse pharmacological effects including anti-inflammatory, anti-oxidative, and anti-tumorigenic properties (Yu et al., 2022). Notably, formononetin has demonstrated inhibitory effects on various types of cancers, such as breast, ovarian, cervical, prostate, gastric, and lung cancers (Aliya et al., 2023; Almatroodi et al., 2023). Studies have indicated that formononetin, by inhibiting EGFR, can serve as one of the candidate drugs targeting TNBC (Lu et al., 2024). EGFR, a tyrosine kinase receptor, plays a pivotal role in the growth regulation of both normal cells and malignant tumor cells. Research has shown that EGFR is highly expressed in various tumor cells, particularly in TNBC, where its overexpression is closely associated with aggressive features such as high invasiveness, increased risk of distant metastasis, and poor prognosis (Kim et al., 2023; Yin et al., 2020). Although there is currently a lack of extensive clinical trial data to support this, formononetin can be used in conjunction with other chemotherapeutic agents and targeted therapies to enhance treatment efficacy (Yu et al., 2020). For instance, when combined with EGFR inhibitors like gefitinib or cetuximab, it can further augment the inhibition of EGFR, ultimately improving the prognosis for TNBC patients. This suggests the therapeutic potential of formononetin in TNBC. Our preliminary research as highlighted the efficacy of formononetin in targeting the Akt/mTOR pathway in TNBC (Zhou et al., 2019).

In this study, we aimed to investigate alternative mechanisms of PCD induced by formononetin, specifically focusing on the role of ferroptosis inhibition Fer-1 in formononetin-induced cytotoxicity. *In vitro* and *in vivo* experiment, our results showed that formononetin inhibited the activity and proliferation of TNBC cells. In addition, our findings revealed that Fer-1 exhibited partial efficacy in mitigating formononetin-induced cell death, suggesting its potential as a therapeutic intervention (Mou et al., 2019). We observed significantly elevated levels of biochemical markers associated with ferroptosis in breast cancer cells following formononetin treatment, indicating an enhanced sensitivity to ferroptosis in the presence of formononetin.

In addition, we demonstrated the therapeutic potential of a combination of mTORC1 inhibition and ferroptosis induction with formononetin in TNBC. Our study provided evidence that formononetin exerted a significant inhibitory effect on the expression of mTORC1, a negative regulator of ferroptosis (Zhang et al., 2021). mTORC1 hinders ferroptosis by facilitating the uptake of cystine through xCT and subsequent synthesis of GSH, while it also directly enhances the synthesis of GPX4 to counteract ferroptosis (Xuan et al., 2022).

Furthermore, the persistent activation of the PI3K/Akt/mTORC1 signaling pathway induces resistance to cancer ferroptosis through the upregulation of SREBP1 (Yi et al., 2020). In recent studies, SREBP1 has been found to play a crucial role in the regulation of ferroptosis by the PI3K-AKT-mTOR signaling pathway. When the PI3K-AKT-mTOR signaling pathway is activated, mTORC1 can inhibit ferroptosis by upregulating the expression of SREBP1(He et al., 2024; Wu et al., 2021; Zhou et al., 2023). This mechanism has been verified in multiple tumor cell lines, indicating that SREBP1 is one of the key downstream targets of the PI3K-AKT-mTOR signaling pathway in regulating ferroptosis.

SREBP1, a pivotal transcription factor that regulates the expression of genes involved in lipid homeostasis synthesis (He et al., 2024), including ATP Citrate Lyase (ACLY), fatty Acid Synthase (FASN), acetyl-CoA carboxylase alpha (ACACA), and SCD (Yi et al., 2020). These genes play significant roles in lipid metabolism, influencing the synthesis and distribution of intracellular lipids, thereby modulating the cellular sensitivity to ferroptosis. Studies have also shown that SREBP1 primarily protects cells from ferroptosis by upregulating the expression of SCD1 (Zeng et al., 2022). SCD1 is an enzyme that catalyzes the conversion of stearoyl-CoA to monounsaturated fatty acids and occupies an important position in lipid metabolism. By increasing the expression of SCD1, SREBP1 promotes the synthesis of monounsaturated fatty acids within cells (Oishi et al., 2017). These fatty acids possess antioxidant properties, capable of reducing the accumulation of lipid peroxidation products, thereby decreasing the sensitivity of cells to ferroptosis.

SCD1 serves as the primary focus of SREBP1, while also functioning as a pivotal enzyme within the MUFA synthesis pathway, thereby governing the modulation of cellular ferroptosis sensitivity (Jaeschke et al., 2021). Experimental manipulation through knockdown and overexpression techniques has substantiated the direct targeting of SREBP1 by formononetin, establishing a significant association with the regulation of lipid peroxide accumulation mediated by formononetin.

**FIGURE 6:**
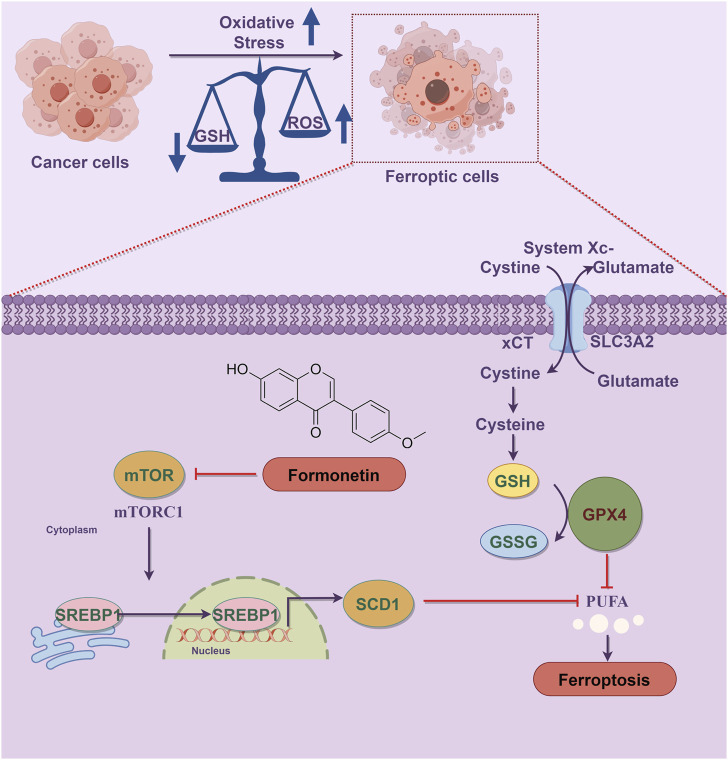
Schematic diagram of the mechanism of formononetin induces ferroptosis in triple-negative breast cancer cells. Ferroptosis is a form of programmed cell death characterized by the accumulation of lethal lipid peroxides due to heightened iron-mediated generation and inadequate removal of reactive oxygen species. Oxidative stress plays a crucial role in the progression of ferroptosis, with the dysregulation between lipid peroxide production and antioxidant defense mechanisms serving as a key initiator of this process. Formononetin intervention led to the inhibition of mTOR1 activity, thereby preventing SREBP1 entry into the nucleus and subsequently reducing the transcription of the SREBP1 target gene SCD1, resulting in PUFA accumulation. Additionally, formononetin directly inhibited xCT activity, decreasing cysteine uptake in breast cancer cells, impairing intracellular GSH synthesis, and diminishing lipid peroxide reduction capacity, ultimately facilitating ferroptosis.

In summary, as demonstrated in Figure 6, our study provides evidence that formononetin possesses the ability to suppress tumor proliferation both *in vitro* and *in vivo* by inducing ferroptosis in breast cancer cells. This effect is achieved through the inhibition of the mTOR/SREBP1/SCD1 axis. Additionally, formononetin primarily promotes ferroptosis by modulating the activity of the xCT/GSH/GPX4 axis, which is mediated by the downregulation of SREBP1 expression. Consequently, targeting SREBP1 may represent a promising and valuable therapeutic strategy for formononetin-induced ferroptosis in the treatment of TNBC. In future studies, further investigation into the regulatory effects of formononetin on mTOR and SREBP1 expression and activity may provide a more comprehensive understanding of its role in ferroptosis.

This study, however, does have certain limitations. Firstly, the *in vivo* experiment only observed the pharmacodynamic effect of formononetin on subcutaneous transplanted tumors in nude mice, without utilizing mTOR inhibition to monitor tumor changes. Secondly, the focus of this paper solely examined the impact of formononetin on ferroptosis, neglecting to investigate the interconnection between ferroptosis and other PCDs mechanisms simultaneously. It is crucial for future research to determine the proportion of ferroptosis in formononetin -mediated breast cancer cell death and to explore the cross talk between various PCDs, as this represents a significant avenue for further investigation.

## Data Availability

The raw data supporting the conclusions of this article will be made available by the authors, without undue reservation.

## Abbreviations

mTORC1, Mammalian target of rapamycin complex1; MDA, malondialdehyde; GSH, glutathione; Fer-1, Ferrostatin-1; ROS, reactive oxygen species; TNBC, triple-negative breast cancer; SREBP1, Sterol regulatory element-binding protein.
