# Supplementary material for: Formononetin triggers ferroptosis in triple-negative breast cancer cells by regulating the mTORC1/SREBP1/SCD1 pathway
Source: Front Pharmacol. 2024 Sep 27;15:1441105. doi: 10.3389/fphar.2024.1441105 (PMC11470441; doi:10.3389/fphar.2024.1441105)
Supplement: Supplementary file 1 [file Table1.DOCX]

# Supplementary data

### Supplementary Table I. Experimental materials and reagents

| **Name** | **Company** | **Batch number** |
| --- | --- | --- |
| **Drugs** |  |  |
| Formononetin | MCE | HY-N0183 |
| RSL3 | Selleck | S8155 |
| Fer-1 | MCE | HY-100579 |
| Torin 1 | Selleck | S2827 |
| Paclitaxel | Selleck | S1150 |
| **Reagents** |  |  |
| Matrigel | Corning | 356234 |
| MDA assay kit | Beyotime biotechnology | S0131S |
| Liperfluo assay kit | DOJINDO | L248 |
| GSH assay kit | Beyotime biotechnology | S0053 |
| ROS assay kit | Beyotime biotechnology | S0033S |
| Iron Assay Kit | DOJINDO | I291 |
| Crystal Violet Staining Solution | Beyotime biotechnology | C0121 |
| CCK8 assay kit | Top science | C0005 |
| DAB horseradish peroxidase color development kit | Beyotime biotechnology | P0202 |
| **Anti-body** |  |  |
| p-mTOR | CST | 5536 |
| mTOR | abcam | ab3876 |
| SCD1 | abcam | ab236868 |
| S6K | abcam | Ab32529 |
| SREBP1 | abcam | Ab28481 |
| xCT | abcam | Ab307601 |
| GPX4 | abcam | Ab125066 |
| Ki67 | abclonal | A20018 |
| DAPI | CST | 4083 |
| **Plasmid transfection** |  |  |
| Lipo8000^TM^Transfection Reagent | Beyotime biotechnology | C0533 |
| SREBP1--overexpression RNA | Gene Chem |  |
| SREBP1-RNAi | Gene Chem |  |
